# Efficacy of exposure in group settings for youth with posttraumatic stress symptoms

**DOI:** 10.1186/s13034-021-00408-z

**Published:** 2021-09-25

**Authors:** Elisa Pfeiffer

**Affiliations:** Department of Child and Adolescent Psychiatry/ Psychotherapie, Ulm University, Steinhoevelstraße 1, 89075 Ulm, Germany

**Keywords:** Trauma, PTSD, Treatment, Group, Exposuref, Youth

## Abstract

**Background:**

Exposure to traumatic experiences is a fundamental part of evidence-based trauma-focused cognitive behavioral treatment (CBT) but in group settings it is discussed controversially among researchers and practitioners. This study aims to examine the individual participants’ stress level during group sessions with exposure and disclosure of traumatic events.

**Method:**

*N* = 47 traumatized youth (*M*_*age*_ = 17.00, 94% male) participated in a group intervention comprising six 90-min group sessions (exposure in sessions 2–5). It is based on trauma-focused CBT principles. The individual stress level was assessed by the participants and group facilitators at the beginning, during, and at the end of every session.

**Results:**

During the sessions including exposure, the stress level of the participants was higher than during sessions without exposure (*Z* = − 3.79; *p* ≤ .001). During the exposure sessions, the participants showed significant changes in stress level (*d* = 0.34–0.87) following an inverse U-shaped trend.

**Conclusion:**

The results show that exposure is feasible within the scope of a trauma-focused group intervention for youth. The further dissemination of trauma-focused group treatments is an important component in the mental health care of children and youth who are traumatized.

## Background

Approximately 16% of children and youth develop posttraumatic stress disorder (PTSD) in the aftermath of exposure to trauma [1]. If left untreated, PTSD becomes a chronic, disabling disease which is associated with severe long-term mental and physical health consequences [2, 3]. There are several empirically supported treatments for children and youth to help alleviate PTSD symptoms [4]. Trauma-focused cognitive behavioral treatment (CBT) approaches enjoy the most empirical support [5] and are recommended in international treatment guidelines [6]. Group trauma-focused CBT has received less attention in child PTSD research despite the numerous advantages of group treatments such as mutual support, group cohesion or cost-effectiveness. A field trial in the aftermath of Hurricane Katrina in New Orleans (*N* = 118) even showed comparable significant results in terms of reductions in PTSD and depression among the participants in “Cognitive Behavioral Intervention for Trauma in Schools (CBITS)” [7] and participants in the individual trauma-focused CBT treatment condition (TF-CBT; 7,8). Notably, attrition rates of treatments with in-session exposure (26.4%) are significantly higher than treatments without in-session exposure (18.9%, *p* < 0.001) [9].

Despite the effectiveness and potential benefits of group trauma-focused CBT, not only researchers but also clinicians are often hesitant when it comes to implementing them [10, 11]. Next to practical reasons (e.g. recruitment of sufficient participants to build a group), a major concern in implementing trauma-focused group treatments is the exposure component which normally includes disclosure of the traumatic experiences in the group. In a recent meta-analytic review of exposure in a group format with adult samples, Barrera et al. [12] summarize three major concerns about in-group exposure: (1) Vicarious traumatization of group members by hearing details of other group members’ traumatic experiences; (2) unhelpful comparisons between group members’ own traumatic experience and those shared in the group, resulting in a biased perception of their experience, and (3) due to limited number of time and sessions in a group format on the one hand, and the high number of traumatic experiences of all group participants that might need to be addressed on the other hand, the group format might be less efficient than individual treatment. None of these claims is backed by research findings but seem nevertheless to be widely advanced by researchers and clinicians alike.

The role of exposure in trauma-focused group CBT for children and youth has not been investigated yet. As most group protocols either exclude in-session exposure [13], conduct exposure in extra-individual sessions (group TF-CBT; [14]) or only briefly touch on them in the group setting (CBITS), we know very little about whether in-session exposure can be successfully administered in trauma-focused group protocols for children and youth. This leaves a crucial gap in the literature.

Hence, this study aims to investigate the feasibility of exposure in a trauma-focused CBT group treatment for youth with PTSD symptoms. The underlying mechanism of exposure, which roots in classical conditioning, is the assumption that through the process of (repeated) confrontation of trauma-related stimuli, which is associated with a strong emotional (e.g., fear, sadness, stress) and physical (e.g., heart racing, trembling) response, habituation of emotional responses with the trauma occurs. The patient experiences evidence that disconfirms dysfunctional cognitions especially related to self-competence and control. Fear activation during exposure is considered a necessary condition for correcting pathological elements of the fear structure in PTSD. The objective of this study is, therefore, to analyze levels of stress (fear activation) during exposure and non-exposure sessions in the trauma-focused group intervention “Mein Weg” (English “My Way”) [15] which is based on trauma-focused CBT principles comprising psychoeducation, relaxation, exposure, and cognitive restructuring. A pilot study [16] and a subsequent randomized controlled trial (RCT; [16]) demonstrated the feasibility and effectiveness of the intervention in reducing PTSD symptoms and depression. Based on current literature on the effectiveness of exposure-based trauma-focused CBTs, I hypothesize that the general stress level is significantly higher during exposure sessions than in sessions without exposure (H1) and that the participants’ stress level will increase significantly and subsequently decrease significantly during exposure sessions (H2).

## Methods

### Study design

This study is part of a single-blind parallel-group RCT (“Mein Weg” versus “usual care”) which was conducted in Germany in 2016–2017 [17]. The “usual care” condition consisted of the regular care in child welfare programs, which is rather diverse (group homes with staff available 24 h/day or shared apartments with care by social workers for several hours a week) but normally don’t include psychotherapy or psychiatric services. If necessary, though, youth are referred to mental health services nearby. The study protocol was approved by the IRB at Ulm University (#176/16) and pre-registered in the German Clinical Trials Registry (#DRKS00010915). All study participants and their legal guardians in the case of minors gave their informed written consent before being enrolled in the study. Participants were recruited between November 2016 and January 2017 in 12 cooperating child welfare agencies in Southern Germany. Specifically trained child welfare staff invited all refugee minors in their agency to the study. At the screening appointments, the study coordinators and child welfare staff informed the youth about the study and assessed the eligibility criteria. Inclusion criteria for study participants were: (A) age 13–21 years, (B) not undergoing alternative behavioural treatment, (C) being able to participate in daily activities such as school, (D) a history of exposure to one or more traumatic event(s), (E) at least moderate severity of PTSD symptoms, (F) basic command of the German language, (G) residency in Germany for at least 6 months, (H) prospect of at least 3 months’ continuation of the current CAW program, (I) no acute suicidality, (J) willingness and ability to attend weekly intervention sessions, and (K) informed consent from the participants themselves as well as the legal guardians in the case of minors.

### Participants

After randomization, *N* = 50 participants were allocated to the “Mein Weg” intervention. In the group compilation phase, *n* = 2 (4%) did not start the intervention for practical reasons in the child welfare program, and *n* = 1 (2%) decided not to start the intervention due to a lack of motivation. After all intervention groups had started, *n* = 10 (22%) participants did not participate in the full format of at least five sessions (reasons: lack of motivation: *n* = 4; 8.51%; alternative treatment: *n* = 1; 2.13% (alternative treatment: medication); high psychosocial stress through a deportation notice: *n* = 1; 2.13%; organizational reasons in the child welfare programs: *n* = 4; 8.51%). Intervention non-completers did not differ significantly from completers in terms of sociodemographic and outcome variables at baseline [16]. All participants who started the intervention (*n* = 47) were included in the statistical analyses. The participants were between 14 and 19 years of age (*M*_*age*_ = 17.00; *SD*_*age*_ = 1.12) and mostly male (*n* = 44; 93.6%). The majority of participants came from Afghanistan (*n* = 25; 53.2%), followed by Syria (*n* = 4; 8.5%), Gambia (*n* = 4; 8.5%), and Somalia (*n* = 3; 6.4%). There were two participants each from Iran and Pakistan as well as one participant each from Senegal, Guinea, Guinea-Bissau, Iraq, and Eritrea.

### Intervention

The “Mein Weg” intervention is a trauma-focused, component-based group intervention, specifically designed for young refugees who experienced traumatic events and subsequently suffer from PTSD symptoms. It is delivered in a format of six weekly 90-min sessions and implemented by two trained group facilitators in groups of two to five participants in the child welfare facility. The manualized intervention comprises elements of trauma-focused CBT and group processing principles. The CBT components include psychoeducation and relaxation (session 1), exposure (trauma narrative) and cognitive restructuring (sessions 2–5), and enhancing safety and future development (session 6) (for more information on the session outline, please see Pfeiffer et al. [17] and Pfeiffer and Goldbeck [15]). Each session starts with a welcome round and a review of the content of the previous sessions and homework, and ends with a positive activity (e.g., playing a game, playing music together). The general group processing principles include sharing experiences, feelings and problems, and mutual support when sharing the trauma narrative. The intervention manual is accompanied by different materials such as a culture-sensitive workbook to reduce potential cultural and linguistic barriers. In the current study, the intervention is delivered by specifically trained and supervised social workers in the participating child welfare programs. Overall treatment fidelity to the manual, as assessed using session checklists, was high with 97% of the content marked as completed [17]. If a participant misses a session, the group facilitator reviews the group session content with the participant individually.

#### In-session exposure

Gradual exposure to traumatic experiences is covered in sessions 2–5. A metaphor (“the wound metaphor”) is used to explain the rationale behind exposure at the beginning of session 2. Each session focuses on a different topic: session 2 ‘My life in my home country’ and ‘My way to Germany’, session 3 ‘My worst experience’ (which could have happened before, during or after the migration), session 4 ‘In Germany–in safety’ and session 5 ‘Letter to a fellow unaccompanied refugee minor’. Participants first draw or write (in their mother tongue, English or German) about the event(s) by themselves with the support of the group facilitator. Afterwards, all participants come together, describe their current level of stress, and then share their narrative with the group. At the beginning of each session, the previous narrations are reiterated either individually or in the group to ensure habituation of the traumatic experiences.

### Measures

#### Participant stress level

A 1-item measure of stress level rated on a 10-point Likert scale (0 = “not stressed at all” to 10 = “extremely stressed”) was introduced to the participants. Participants were instructed to take into account both emotional and physiological stress. The group facilitator made sure that the youth understood the concept and ratings. Participants were asked several times during each session (at least three times) to estimate and report their stress level to the group. The group facilitator also rated each participant’s emotional and physiological stress level. The proxy rating by the facilitator and the participant’s self-report were combined into an overall score at three fixed measurement time points (at the beginning of the session (T1), during the session (T2), and at the end of the session (T3)) and documented in the session protocol. The stress level of the first session was only rated by the group facilitator.

#### Participants’ motivation

The group facilitator rated the group’s overall motivation for each session on a 10-point Likert scale (0 = “not at all motivated” to 10 = “highly motivated”) and documented it in the session checklist.

### Statistical methods

Descriptive analyses were carried out for demographic data and the stress level for each session across all measurement time points. Due to the non-normality of the data, non-parametric analyses were conducted. The Friedman Test was used to analyze differences within exposure sessions across the different measurement time points. Wilcoxon Tests were used to investigate differences between exposure (sessions 2–5) and non-exposure (sessions 1, 6) sessions for all measurement time points and as post-hoc analysis to test for differences between time points in each exposure session. Dependency of the sample is assumed in both analyses.

There was only one missing value within the sessions [in session 2 at the end of the session (T3)] but several missing values between sessions as participants missed entire group sessions (session 1: 15%; session 2: 12%; session 3: 15%; session 4: 26%; session 5: 23%, and session 6: 23%). The intervention completers [participants who participated in every session (*n* = 31; 66%)], non-completers [participants who dropped out during the intervention (*n* = 8; 17%)] and participants who missed single sessions (*n* = 8; 17%) did not statistically significantly differ regarding their age (*p* = 0.319), sex (*p* = 0.455), duration of time spent in Germany (*p* = 0.316), baseline scores of PTSD (*p* = 0.523), and depression (*p* = 0.524). Missing data was not therefore imputed in the primary analyses.

However, as sensitivity analyses, all analyses were repeated and missing values between sessions were replaced by employing multiple imputation [18]. The attendance (yes/no) of the participant as well as the overall group’s motivation in each session were added as predictors of the model. As non-normally distributed variables may introduce bias, the individual variables were first transformed via log-transformation into approximate normality before the imputation process was conducted, as suggested by Sterne et al. [19] and Royston [20].

The significance level of all analyses was set at *p* = 0.05 (2-tailed). Effect sizes (Cohen’s d) were calculated for comparisons. All analyses were performed using SPSS Version 26.

## Results

There were no significant differences between exposure sessions and non-exposure sessions at T1 (*Z* = − 0.14; *p* = 0.889) and at T3 (Z = − 1.87; *p* = 0.062), but the stress level at T2 was significantly higher in exposure sessions than in non-exposure sessions (*Z* = − 3.79; *p* ≤ 0.001) (see Table 1), indicating a higher stress level during exposure.

**Table 1:** Descriptives and test statistics of stress levels across all sessions per measurement time points

Measurement time point	Exposure^a^ sessions		Non-exposure^b^ sessions			
	*M (SD)*	*Md*	*M (SD)*	*Md*	*Z*	*p*
T1	3.24 (1.97)	3.50	3.17 (1.95)	3.50	− 0.14	0.889
T2	5.42 (1.74)	5.50	3.34 (2.12)	2.75	− 3.79	≤ 0.001
T3	3.25 (1.91)	3.50	2.39 (1.74)	2.00	− 1.87	0.062

T1 = at the beginning of the session, T2 = during the session; T3 = at the end of the session

^a^Sessions 2, 3, 4, and 5

^b^Sessions 1 and 6

**Fig. 1:**
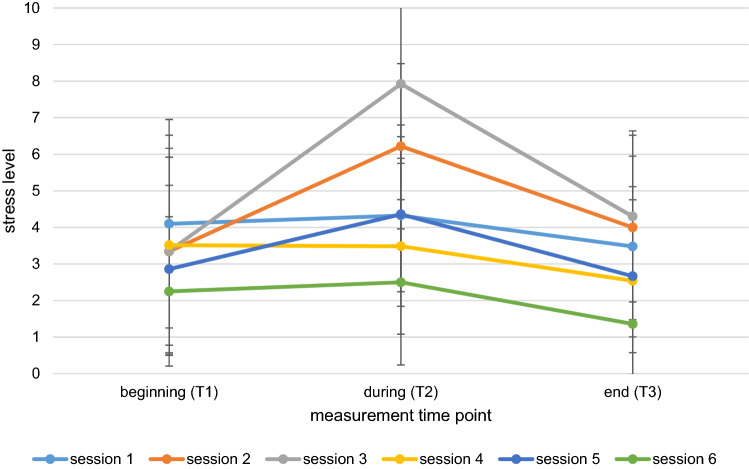
Mean scores of participants’ stress level for each session from beginning (T1) to end (T3)

A significant main effect of time emerged within all sessions (*p* ≤ 0.001) (see Table 2, Fig. 1). Regarding exposure sessions, the stress level significantly increased from T1 to T2 with medium (session 2: *d* = 0.65; session 5: *d* = 0.55) to high (session 3: *d* = 0.87) effect sizes, except session 4 (*p* = 0.997). Similar effect sizes but in a reversed direction were observed across all sessions for T2 to T3, with significant lower levels of stress at T3 compared to T2 [a small effect size for session 4 (*d* = 0.35); medium effect sizes for sessions 2, 3 and 5 (*d* = 0.53; *d* = 0.70; *d* = 0.45 respectively)]. A comparison of T1 to T3 did not reveal any significant differences in session 3 (*p* = 0.083) and session 5 (*p* = 0.472). For sessions 2 (*p* = 0.047) and 4 (*p* = 0.001), differences between T1 and T3 were significant with small to medium effect sizes (*d* = 0.23; *d* = 0.38, respectively). In non-exposure sessions the stress level significantly decreased between T1 and T3 and between T2 and T3 with small to medium effect sizes (*d* = 0.27–0.40). However, no U-shaped trend was observed (Fig. 1). In session 6 the stress level is notably low for the entire session and stress levels are comparably high for session 1 at T1.

**Table 2:** Descriptives and test statistics between measurement time points for each exposure session

Session	T1		T2		T3		T1-T2			T1-T3			T2-T3			Total	
	*M (SD)*	*Md*	*M (SD)*	*Md*	*M (SD)*	*Md*	*Z*	*p*	*d*	*Z*	*p*	*d*	*Z*	*p*	*d*	*Χ*^*2*^(2)	*p*
Session 1 (*n* = 46)	4.10 (2.85)	4	4.33 (2.48)	5	3.68 (2.47)	3	− 0.94	0.345	0.1	− 1.97	0.048	0.27	− 2.98	0.003	0.39	13.08	≤ .001
Session 2^a^ (*n* = 41)	3.34 (2.83)	3	6.22 (2.26)	7	4.0 (2.52)	4.5	− 5.25	≤ .001	0.65	− 1.99	0.047	0.23	− 4.95	≤ .001	0.53	51.25	≤ .001
Session 3^a^ (*n* = 40)	3.35 (2.58)	3	7.92 (2.18)	9	4.3 (2.34)	4.5	− 5.39	≤ .001	0.87	− 1.74	0.083	0.33	− 5.53	≤ .001	0.7	63.88	≤ .001
Session 4^a^ (*n* = 35)	3.51 (3.01)	3	3.49 (2.41)	4	2.54 (2.57)	2	− 0.03	0.977	0.01	− 3.23	≤ 0.001	0.38	− 2.37	0.018	0.35	15.61	≤ .001
Session 5^a^ (*n* = 36)	2.86 (2.29)	3	4.36 (2.13)	4	2.67 (2.10)	2	− 3.96	≤ .001	0.55	− 0.72	0.472	0.07	− 5.01	≤ .001	0.45	43.39	≤ .001
Session 6 (*n* = 36)	2.25 (2.05)	2.5	2.50 (2.26)	2	1.36 (1.38)	1	− 1.21	0.226	0.15	− 3.42	0.001	0.4	− 4.19	0.001	0.4	23.76	≤ .001

T1 = at the beginning of the session, T2 = during the session; T3 = at the end of the session

^a^exposure sessions

**Fig. 2:**
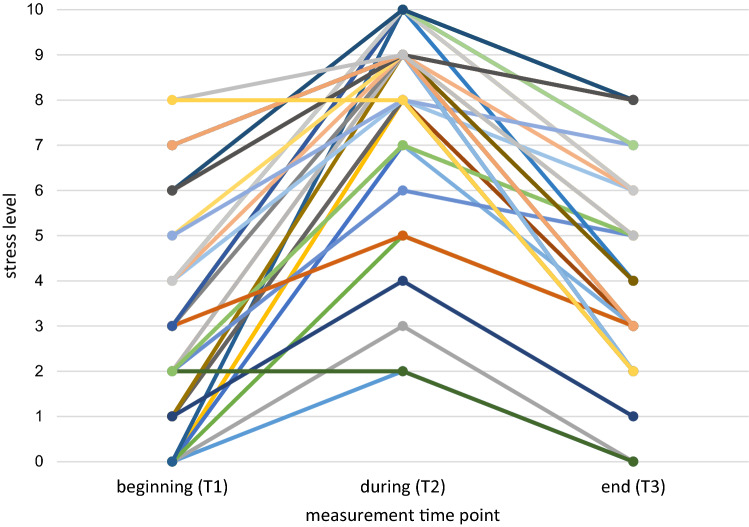
Stress level of participants at the beginning, during, and at the end of session 3. Each line represents a participant (*n* = 47)

In addition to these analyses of mean scores across all participants, Fig. 2 shows the individual stress level ratings of participants in session 3, the session with highest stress levels during the session. Figure 2 supports the findings that participants’ stress levels increased between T1 and T2 and decreased between T2 and T3. Only two participants did not present any increase in stress level between T1 and T2.

In terms of sensitivity analyses, only one minor difference emerged when re-calculating all analyses with imputed data: the stress levels were significantly different across all measurement time points between exposure and non-exposure sessions (*p* ≤ 0.001).

## Discussion

This is the first study to investigate the inclusion of exposure in trauma-focused CBT group treatment for youth who experienced traumatic events and subsequently developed PTSD symptoms. Hypothesis 1 was confirmed as stress levels were significantly higher during exposure sessions, compared with non-exposure sessions. This is consistent with previous studies in adults which demonstrated the effectiveness of CBT group formats in terms of in-session exposure and disclosure [21]. Comparisons of stress level ratings during exposure sessions yielded intriguing results and confirmed hypothesis 2. Large effect sizes for in-session stress level change (T1 to T2, and T2 to T3) were found for sessions 2 and 3. This is in line with the manual that suggests that exposure to traumatic events is most intense in these sessions due to their content. From a large body of research on refugees we know that most traumatic events happened in the home country or whilst on the move [22]. This explains why many traumatic experiences were addressed in session 2. In session 3, the worst event is specifically addressed which might explain the increase in reported stress. In session 4, no U-shape trajectory of the stress level was detected, only a decrease in stress throughout the session. There are several potential explanations for this finding: Session 4 addresses life in the host country (Germany) compared with life in the home country. Consequently, participants may have predominantly focused on positive factors in the host country (such as provision of basic care through child welfare program or schooling) but discussed differences to their home country (which may have included aversive stimuli that elicited stress due to traumatic events in home country) in the group discussion which took place after rating (T2). Another possible explanation could be that the overall effect sizes for increase and decrease were small in this session. This would seem to imply that session content may not have resulted in any major exposure overall.

The results for individual in-session change (Fig. 2) were particularly encouraging for the field as this graph shows that almost all participants (independent of symptoms, traumatic experiences and so forth) were able to experience an increase and decrease in stress level, meaning that they seem to have successfully confronted their traumatic experiences.

It is important to note that the stress level significantly decreased in non-exposure sessions as well. In session 1 this could be explained by the high stress level at the beginning of the session indicating participants’ general nervousness and anxiety about starting a trauma-focused intervention. In session 6 general levels are very low indicating a floor effect. At the end of session 6 the group held a graduation party which might explain the very low scores.

### Limitations and future research

Several limitations which might impede the generalizability of findings need to be addressed. Firstly, and most importantly, this study does not allow any conclusions to be drawn about the habitation process as group members were neither systematically asked to report their stress level during the revision of previous narrations at the beginning of the sessions, nor was this documented by the group facilitators. Thus, the mechanism of exposure could only be observed for the increase and decrease in stress during the specific content (in exposure sessions the narration) of each session. Future studies should seek not only to replicate and build on the present study but also to assess more systematically the stress level at more measurement time points during each exposure session (e.g., specifically during the revision of the narration of prior sessions at the beginning of every exposure session).

Secondly, this secondary analysis comprised a rather small sample of participants which is still nonetheless comparable in terms of sample size to other studies on group trauma-focused treatments with youth [23] or adults [12]. The gender distribution with 93.6% of the participants being male, does not allow for any conclusions regarding female participants, though. The imbalance in the gender distribution can be explained by the general characteristics of the unaccompanied refugee minors population in Germany between 2016 and 2017, who were mostly male. All participants in the study were unaccompanied refugee minors, which might limit generalizability of the findings for children and youth without a migration background. In fact, rates of traumatic experiences, PTSD, and depression among refugee minors are higher than in western samples (e.g., [24]). However, research has shown that refugees benefit just as much from established trauma-focused treatments [25, 26]. This may indicate that they may constitute an especially vulnerable and burdened population but one which is not that different in terms of treatment response.

Thirdly, the study protocol did not include the systematic documentation of how much content of the trauma narrative was actually disclosed and shared in the group discussion in each exposure session. Solely non-standardized reports of group facilitators in supervision gave the impression that almost all participants wanted to share details about their traumatic experiences as they often appreciated the social support from peers and made the valuable experience that other group members had had similar experiences. Experiences and disclosure of sexual abuse are especially associated with feelings of shame and guilt [27] and there might be an unwillingness to share them in a group setting. However, group facilitators reported that these events were shared as well by naming the event and some context (where and when it happened), instead of disclosing many details. Recent research suggests that this brief exposure might elicit a similar activation of the traumatic event resulting in stress and subsequent habituation [28]. Altogether important group processes, such as social support, might come into play during disclosure of traumatic events within a group discussion. Future research needs to investigate group mechanisms and dynamics more closely.

Fourthly, it is important to note that in the first session the stress level rating was probably determined solely by the group facilitator as the stress level measure was not introduced until session 2. It is possible, however, that group facilitators asked the participants about their current stress level in session 1 already, as this was discussed and emphasized in training and supervision.

Fifthly, as this study didn’t include an active control group (e.g., a non-exposure CBT group treatment), other potentially stress-related circumstantial factors, effects of time or order of the intervention, and carry-over effects cannot be excluded. Future research might not only aim at replicating these findings with a comparison with a (trauma-focused) non-exposure CBT group treatment, but also with an individual trauma-focused treatment, such as TF-CBT [8]. Compared with TF-CBT, “Mein Weg” only addresses one worst event, participants spend less time with the trauma narrative (regarding number of sessions and length of session), there is no joint caregiver session and the treatment is delivered by group facilitators who normally have limited experience with exposure and no general CBT training. The gradual exposure in an individual setting might thus be more extensive resulting in a higher stress level during exposure. So far, only one study compared an evidence-based group and individual treatment, but the stress level was neither assessed nor compared [9].

Sixthly, caregivers play an important role in individual trauma-focused treatments [29]. For some participants the group facilitators were also their caregivers of choice and participants were regularly invited to do the “Mein Weg” homework (e.g., read psychoeducation or practice relaxed breathing) with their caregivers, but future implementations of “Mein Weg” could address the pivotal role of caregivers more by implementing caregiver sessions.

Lastly, although it is a major strength of this study that the stress level was assessed directly in the session and evaluated by the participants themselves and the group facilitators, independent and potentially more objective measurements of stress might have generated additional valuable information. Early studies on trauma-focused treatments employed methods such as ratings of facial expressions and coding of videotaped sessions by independent raters [30]. Future research should also take neural and psychophysiological markers into account [31].

### Clinical implications

The trauma-focused group intervention “Mein Weg” was specifically designed and evaluated for young refugee minors with PTSD symptoms. So far, the intervention was implemented in child welfare programs, schools and in a clinical setting (during the developmental phase of the intervention). Hence, if sufficient training and supervision is provided, the intervention can be implemented in various other settings such as the juvenile justice system as well. Regarding the target group, however, the intervention can only be implemented with youth who report traumatic events and a migration history as especially the trauma narrative sessions focus on trauma and the migration process. For youth with PTSD symptoms without a migration history, other evidence-based treatments with a similar theoretical background and program content, such as CBITS, can be implemented.

Since we could not identify any harm to participants that could be traced back to the “Mein Weg” intervention [16, 17], in-session exposure with an increase and decrease in stress among youth who experienced traumatic events seems to be feasible. Hopefully, this will help to refute practitioners’ preconceived ideas about using exposure in a group setting. The findings of this study might serve to motivate practitioners who are reluctant to implement group programs, especially those with in-session exposure, to undergo training in this kind of treatment. In settings in which clinicians’ time and other resources for individual exposure treatment are limited, the implementation of exposure-based group treatments might even increase the number of patients who could receive and benefit from treatment.

Moreover, this intervention was carried out by trained and supervised social workers who are oftentimes referred to as “lay counsellors” as they do not have specific CBT or mental health training. This is a common approach in several trauma-focused individual treatments such as narrative exposure therapy (NET) [32] and group treatments for children such as group-based TF-CBT [33]. Especially in settings that lack trained mental health care professionals, the training and supervision of lay counsellors might ensure a broader dissemination of treatments and access for more children and adolescents in need.

## Data Availability

The datasets used and/or analysed during the current study are available from the corresponding author on reasonable request.
